# Risk and the Republican National Convention: Application of the Novel COVID-19 Operational Risk Assessment

**DOI:** 10.1017/dmp.2020.499

**Published:** 2021-03-25

**Authors:** David Callaway, Jeff Runge, Lucia Mullen, Lisa Rentz, Kevin Staley, Michael Stanford, Crystal Watson

**Affiliations:** 1 Atrium Health, Carolinas Medical Center, Charlotte, NC, USA; 2 Biologue, Inc., Chapel Hill, NC, USA; 3 Johns Hopkins Center for Health Security, Baltimore, MD, USA; 4 Department of Environmental Health & Engineering, Johns Hopkins University Bloomberg School of Public Health, Baltimore, MD, USA; 5 Novant Presbyterian, Charlotte, NC, USA; 6 Charlotte-Mecklenburg Emergency Management, Charlotte, NC, USA; 7 Mecklenburg EMS Agency, Charlotte, NC, USA

**Keywords:** COVID-19, mass gathering medicine, Republican national convention, risk management

## Abstract

The United States Centers for Disease Control and Prevention and the World Health Organization broadly categorize mass gathering events as high risk for amplification of coronavirus disease 2019 (COVID-19) spread in a community due to the nature of respiratory diseases and the transmission dynamics. However, various measures and modifications can be put in place to limit or reduce the risk of further spread of COVID-19 for the mass gathering. During this pandemic, the Johns Hopkins University Center for Health Security produced a risk assessment and mitigation tool for decision-makers to assess SARS-CoV-2 transmission risks that may arise as organizations and businesses hold mass gatherings or increase business operations: The JHU Operational Toolkit for Businesses Considering Reopening or Expanding Operations in COVID-19 (Toolkit). This article describes the deployment of a data-informed, risk-reduction strategy that protects local communities, preserves local health-care capacity, and supports democratic processes through the safe execution of the Republican National Convention in Charlotte, North Carolina. The successful use of the Toolkit and the lessons learned from this experience are applicable in a wide range of public health settings, including school reopening, expansion of public services, and even resumption of health-care delivery.

**“That we may so act, we must study and understand the points of danger.”**

**President Abraham Lincoln, Republican**

The novel severe acute respiratory syndrome coronavirus 2 (SARS-CoV-2) and the disease it causes, coronavirus disease 2019 (COVID-19) resulted in a pandemic that created multiple, complex integrated challenges for health care, public health, and emergency management. The standard approach to Emergency Preparedness is to Prevent, Protect, Mitigate, Respond, and Recover. However, in times of novel or emerging threats, evidence is often scarce and data are frequently so raw as to limit the ability to make objective recommendations. In these times, it is critical to draw on expert opinion and past experience to create adaptive, objective, decision-making tools.

Our team was tasked with creating an operational medical plan for the Republican National Convention (RNC), a designated National Special Security Event (NSSE) and mass gathering, during the highly infectious novel COVID-19 pandemic. Planning a mass gathering event during a national state of emergency related to the COVID-19 pandemic presented several complex health, medical, operational, public policy, economic, and political risks. We faced a totally novel, poorly understood virus with unclear transmission dynamics.^1^ Pandemic models had large margins of error limiting our ability to predict community disease prevalence or anticipated hospital resource use.^2,3^ Existing public health guidance provided only broad, population-based recommendations that did not have robust supporting data.^[Bibr r4]^ And, there existed no regimented process by which to consolidate the impact of implementing multiple mitigation strategies. In short, existing guidance provided little objective data to conduct objective risk reduction.

We identified The Johns Hopkins University (JHU) Operational Toolkit (Toolkit) as an objective risk assessment process developed by Global Health and Infectious Disease experts. This toolkit underwent an internal validation process by the Johns Hopkins Center for Health Security to ensure the weighting of risks and mitigation measures were both accurate and comprehensive. The toolkit was then submitted to field tests with a select group of business leaders, allowing for a review process before its publication. Furthermore, our team determined that each individual component and the collective process was consistent with lessons learned from prior pandemics and aligned with current public health guidance. With this understanding, we applied the Toolkit to a complex mass gathering with national security, political, and public policy implications.

## The COVID-19 Risk Environment

Risk cannot be eliminated, but it can be mitigated. Robust risk assessment and management tools can reduce risk of disease spread during mass gathering events. The foundation of any COVID-19 mitigation strategy requires understanding the underlying hazard, identifying potential amplifying risks, articulating actions that reduce risk, and creating an iterative process for modifying planning as new recommendations emerge. In addition, for political, religious, or social gatherings of high importance, risk perception, and risk tolerance are influential subjective planning modifiers. As such, planners must incorporate risk communication strategies into the management process to ensure leaders and participants can make an accurate assessment of their personal risks, understand the measures that are being taken operationally to reduce risk, and understand the measures that individuals can take to reduce risk.

The United States Centers for Disease Control and Prevention (CDC) and the World Health Organization broadly categorize mass gathering events as high risk for amplification of COVID-19 spread in a community due to the nature of respiratory diseases and the transmission dynamics.^5^ During this pandemic, the JHU Center for Health Security developed a novel risk assessment and mitigation tool for decision-makers to assess SARS-CoV-2 transmission risks that may arise as organizations and businesses hold mass gatherings or increase business operations.^6^ The JHU development team also contributed to numerous WHO risk assessments designed for broader population health application and specific mass gathering events. During the planning for the RNC, the JHU product was the only publically available toolkit designed specifically for businesses or mass gatherings, and most applicable for the RNC context. The RNC COVID-19 Cross Functional Team (CFT) used the JHU Operational Toolkit for Businesses Considering Reopening or Expanding Operations in COVID-19 (Toolkit) to aid in medical and public health planning for the 2020 RNC.^7^

## The Risk Assessment Process

**Figure 1.**
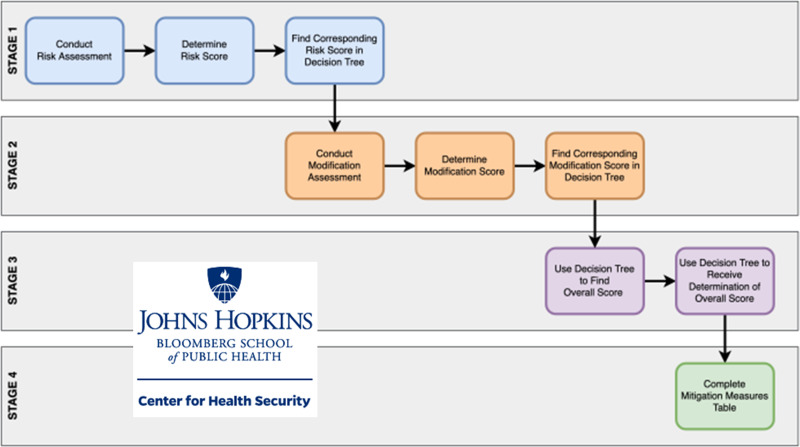
Toolkit utilization process.

The Toolkit quantifies the likelihood that a business or event will amplify community transmission of COVID-19. It also provides a regimented process for identifying risk modification and mitigation measures that organizations can implement to determine a final risk score for the business in light of their inherent risks related to their operations and their efforts to reduce such risks (Figure 1). As part of the internal validation process, the developers of the Toolkit conducted the risk assessment process for numerous business sectors and events to ensure all considerations and potential risks were included in the assessment. Questions posed in the Toolkit to measure risk and the ability to modify or reduce the risk were weighted by experts at JHU in accordance to the relative importance each activity or measure had in contributing to risk or risk reduction. These weightings aligned with evidence-based justifications on COVID-19 transmissions risk and preventative measures, and were consistent with other assessment tools, such as the WHO risk assessments. The 10-member CFT team (Table 1) used a modified Delphi process with the Toolkit to craft consensus risk assessment and mitigation recommendations.

**Table 1.** Cross-Functional Team Membership

1. Emergency medicine expert
2. Specialty advisor for infectious disease
3. Specialty advisor for laboratory medicine
4. Public Health officer
5. Emergency Medical Services
6. Event medicine nursing leader
7. Emergency management representatives (health system and local government)
8. Law enforcement
9. Facility and engineering expert
10. Event planning representative

### Stage 1: Risk Assessment

**Figure 2.**
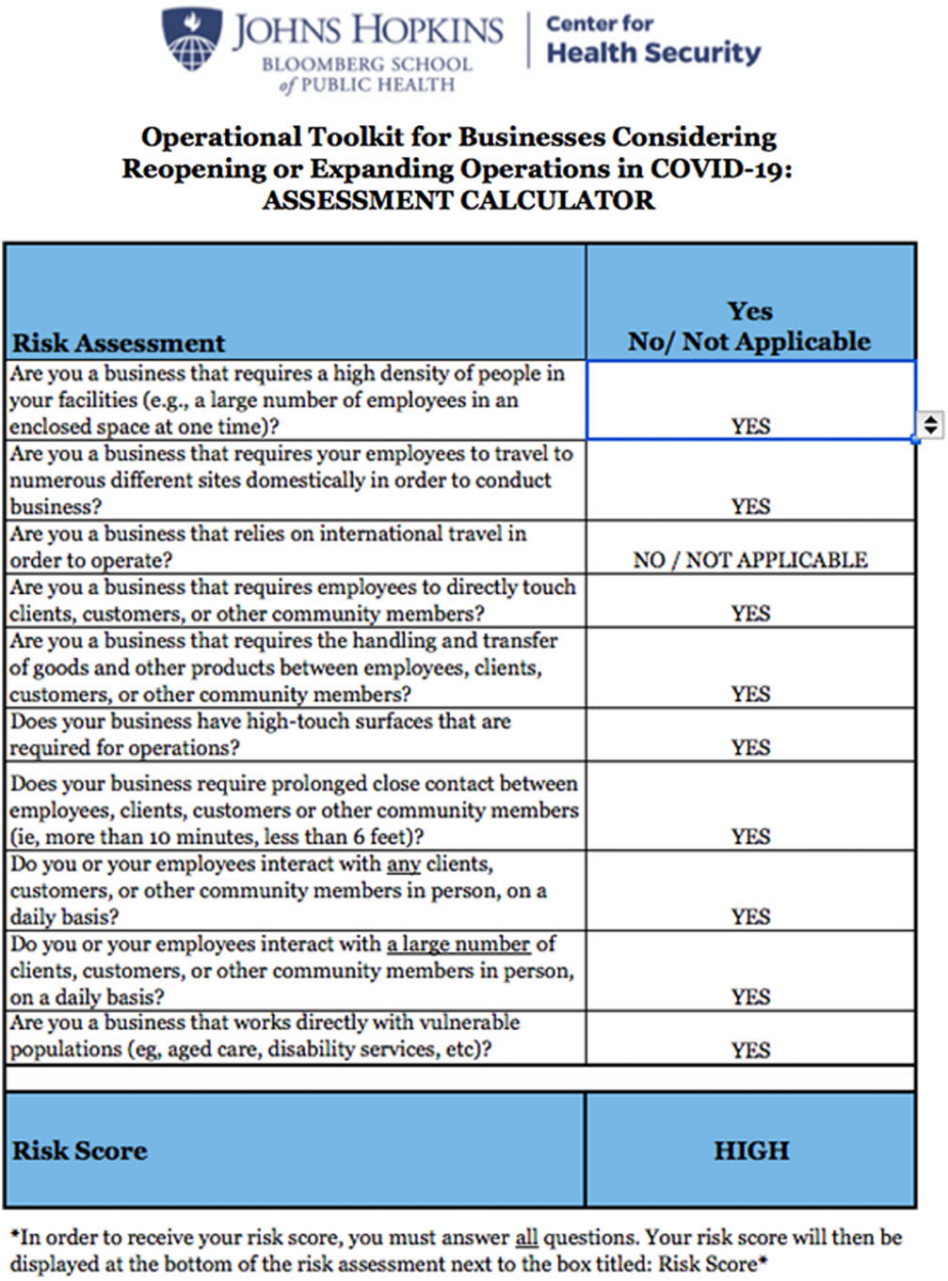
Initial risk assessment.

The initial assessment identifies the inherent risk of an event without intervention and determines the risk score (Figure 2). A critical first step is documenting the planning requirements; in this case, a “full, in-person event” with an estimated attendance of 7000-20,000 people held in a 19,000 person capacity indoor arena. The 4-day NSSE required people to travel from around the country; prohibited a move to online engagements; hosted a variety of large, indoor in-person events; and, within the parameters above, would not allow for recommended 6-foot physical distancing for the bulk of events. The CFT anticipated high incidence of shared surface contact and interpersonal exchange of goods (eg, banners, information, food, etc.) in addition to close person-to-person interactions.

The CFT unanimously assessed that the NSSE posed a HIGH risk of COVID-19 amplification in the community.

### Stage 2: Modification assessment

**Figure 3.**
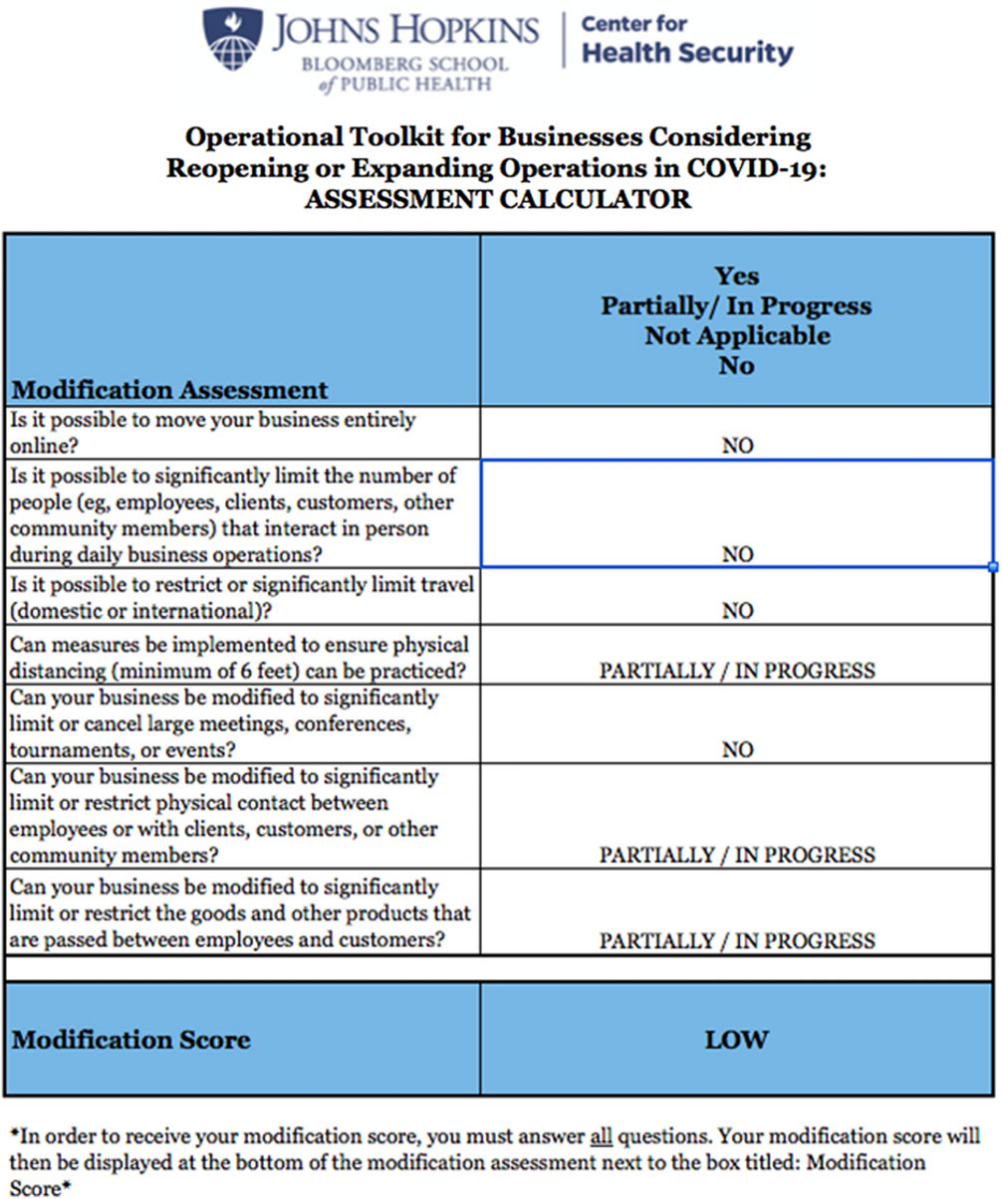
Risk modification assessment.

The modification assessment calculates an updated risk score based on changes to event operations (Figure 3). The JHU subject matter expert team weighted the questions based on qualitative judgments about the relative contribution of each question to reducing the quality and quantity of the event-related interactions that could lead to transmission of COVID-19. Although the level of evidence for question weighting is “expert opinion,” the data-informed tool does reduce potential subjective bias on the part of the evaluation team using the Toolkit.

The Toolkit modification scores range from low (ie, little ability to modify) to high (ie, significant ability to modify operations). From a planning standpoint, a HIGH score is desired to allow for greater risk reduction. Based upon initial RNC planning restrictions, the CFT unanimously assessed that the initial modification score was LOW

### Step 3: Determination of Risk-based on Overall Score

The Toolkit provides 5 overall scores, ranging from Very Low to Very High. No numerical weight is attributed to these scores as it is impossible to precisely quantify the risk of a business or event due to numerous and constantly changing variables, such as individual risks of participants, epidemiological context, and compliance of mitigation measures. However, a score of VERY HIGH can only be achieved if the original risk of the business or event is considered high and little to no modifications are in place to reduce the risk. The CFT determined that the RNC was HIGH risk with a LOW modification score. As a result, the Toolkit assessed the overall risk of COVID-19 amplification in the community as VERY HIGH.

### Step 4: Mitigation Measures

The previous steps focus on the health risks at an operational level and the event modification measures that could reduce overall risk. The mitigation measures focus on decreasing individual risk by promoting personal safety measures. While the mitigation measures do not contribute to the overall score, they should be considered as the foundational element on which planners build a risk and safety strategy. The CFT reviewed the Toolkit mitigation measures and integrated them with an expanded set of additional potential options, such as robust pre-event and daily COVID-19 testing, digital contact tracing technology, and advanced environmental engineering (eg, air filtration systems). The absence of data for each of these more aggressive interventions limited the CFT’s ability to calculate a quantifiable risk reduction value or determine a cost-benefit analysis of the recommendations.

### Stage 5: Revision of Planning Assumptions

**Figure 4.**
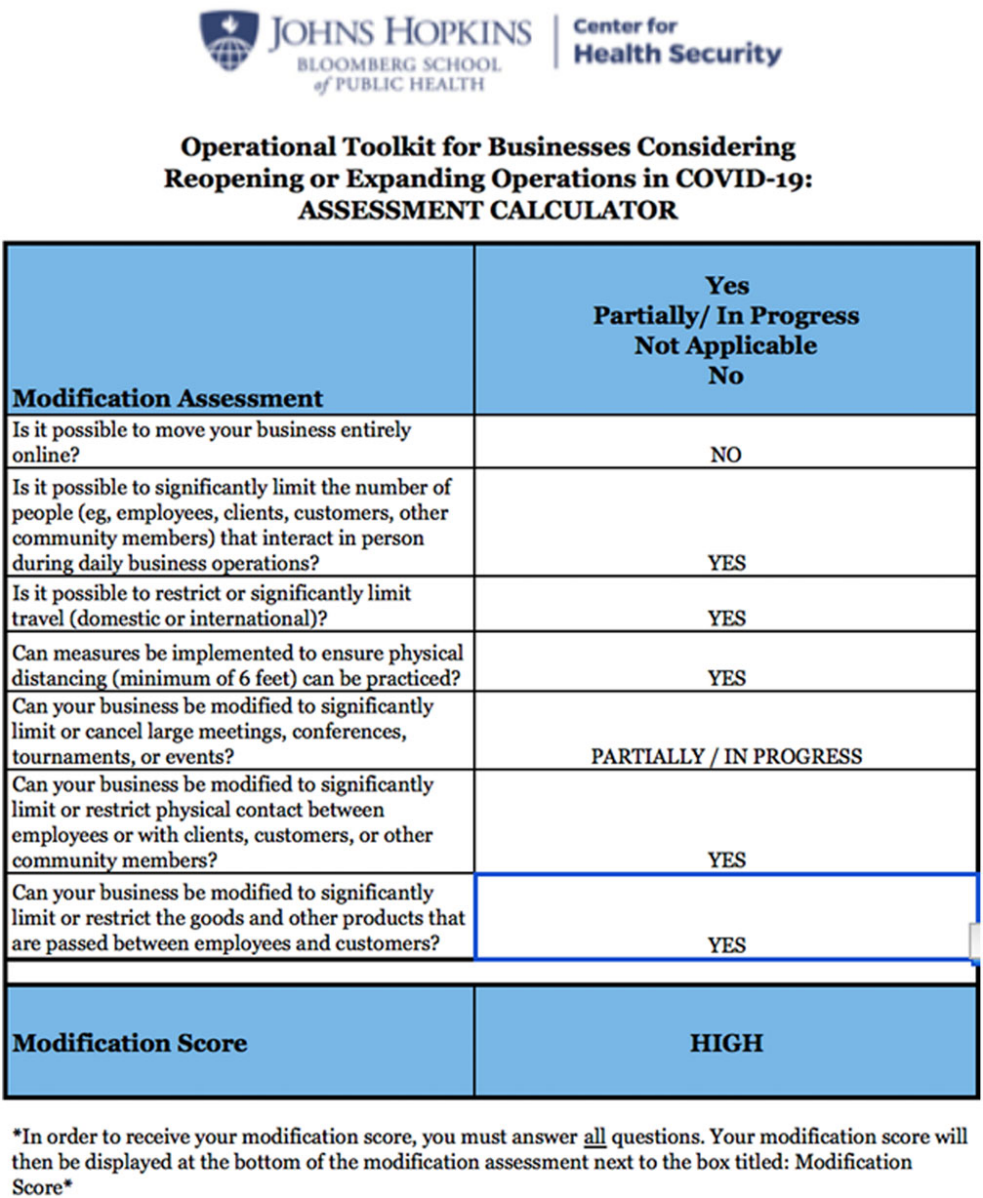
Updated risk modification score.

The CFT added a fifth stage to allow for a more adaptive planning process. In Stage 5, the CFT eliminated the original planning restrictions and completed Stage 2 in an iterative, in-person session. Figure 4 demonstrates an updated modification score that reflects a course of action limiting the number of RNC participants. The downstream impacts of fewer attendees are less travel, an ability to engineer space to allow for physical distancing, and the ability to craft meeting spaces that align with public health recommendations. The impact of reduced participants had broad operational impact and changed the modification score to HIGH. That singular decision to reduce number of participants moved the overall RNC event risk to MODERATE. This rating does not account for use of the advanced mitigation options noted above that further reduced community risk.

The CFT provided event planners with multiple courses of action that articulated baseline COVID-19 transmission risk and various interventions to reduce overall risk. These recommendations allowed event planners to have an informed conversation about risk tolerance and develop a realistic policy surrounding the viability holding the RNC mass gathering event.

Despite the high-profile political nature of the RNC, there were very few hurdles to using the Toolkit. A key to this success was an early iterative process where CFT members articulated individual and agency planning assumptions, consolidated known data surrounding COVID-19 transmission, and created a transparent communication process.

## Planning Hurdles

The 3 main general planning hurdles that the Toolkit helped to overcome were:

1. *Operational and Political Constraints*

The initial, non-negotiable planning assumptions included no mandatory masking, no restrictions on the numbers of participants, and no mandated physical distancing. Every member of the planning team understood that these requirements ran counter to CDC recommendations and would preclude a safe convention. The scientific rigor of using an objective risk assessment tool and engaging nonmedical professionals, such as engineers and human-centered design experts, was critical for creating an unassailable risk assessment process. The fact that the team used an external assessment tool and found universal agreement on overall risk score was vital for communicating recommendations to the RNC leadership team.

1. *Variable Definitions of Risk*

The CFT began the process by identifying the categories of risk as individual health, community health, economic impact of resurgence or event cancelation, political, and social disruption. Each category of risk is necessarily interconnected. For some, such as economic or political risk, very little science is available to guide decision-making. Based upon open-source information and current events, the CFT unanimously agreed that COVID-19 amplification would create high risk of economic, political, and social disruption. With this agreement, the CFT was able to focus on using the Toolkit to quantify individual and community health risk.

1. *Quantifying the Financial Cost of Risk Reduction*

One of the great balancing acts of risk mitigation is aligning the cost of an intervention with the relative impact of that intervention. In this case, reducing the transmission of COVID-19 was paramount and linked to the health, economic, social, and political risk calculations. There was no way to quantify the incremental risk reduction for each advanced intervention. Fortunately, robust funding was available.

## Preliminary Outcomes

The deployment of the JHU Toolkit was an operational and quality assurance decision. Publically reported data revealed that using the Toolkit allowed the team to identify four (4) COVID-19-positive individuals before entry into the NSSE and engagement with other attendees. Since the conclusion of the RNC, there are no reports of COVID-19 transmission from the RNC.

## Keys to Success

To our knowledge, this was the first large-scale application of the Toolkit. The post-hoc analysis produced several operational recommendations for successful use of the Toolkit.

The first consideration is ensuring multi-specialty representation in the cross-functional team to develop the most comprehensive risk assessment. Diverse representation, including nonmedical personnel, facilitates a more dynamic iterative process, reduces professional blind spots, and strengthens validation of recommendations.

The second recommendation is to develop a process for acquisition, analysis, and dissemination of data. During emerging threats, there is often so much data that analysis and transformation of data to information is paralyzed. During periods like these, focused empiricism is a useful strategy, enabling rapid guideline and process development that can be tested and evolved in near real time. The challenge lies in determining valid sources of information and vetting the information while remaining open to nontraditional sources of data. One example was the emerging data on novel air filtration systems. The traditional medical literature had little information on these systems. Fortunately, the CFT engineer had both deep experience with air filtration, knowledge of the cutting-edge research on the topic, and most importantly, understood the valid sources of published data.

The third key to success is to conduct familiarization training on toolkit, then ensure anonymous completion of the Toolkit to reduce any potential social influence. The team leader should consolidate CFT responses and review for consensus. The entire team should then determine whether to revise assumptions. Critically, this team ran multiple trials of the toolkit with a variety of initial planning assumptions. This allowed the team to offer leadership multiple courses of action with objective risk profiles.

## Conclusions

The multi-specialty application of the Toolkit demonstrated a novel process to objectively assess initial risk, evaluate impact of interventions, and rationally advise nonmedical decision-makers. The lessons from this planning process can inform public policy-makers determining re-opening strategies for businesses, schools, and health systems. There are several critical observations. First, the planning team must acknowledge that risk can be mitigated not eliminated. Second, the team must agree upon the definition and core components of the risk to be addressed. Third, building the correct team with broad stakeholder representation is critical to maximize information acquisition, ensure unity of purpose, maintain agreement on planning assumptions, and communicate cohesive recommendations. Finally, during emerging threats, evidence often challenges previous assumptions or recommendations. The planning process must allow for a continuous assessment and refinement of the mass gathering safety plan to integrate new evidence and data.
